# Efficacy and safety of alternative-level laminoplasty vs. all-level laminoplasty: a systematic review and meta-analysis

**DOI:** 10.3389/fsurg.2025.1629037

**Published:** 2025-07-14

**Authors:** Bin Zheng, Ke Ma, Zhenqi Zhu, Haiying Liu

**Affiliations:** ^1^ Spine Surgery, Peking University People's Hospital, Beijing, China; ^2^ Orthopedics Department, Huailai County Hospital, Zhangjiakou, Heibei, China

**Keywords:** laminoplasty, all-level, alternative-level, cervical spondylotic myelopathy, cervical canal volume

## Abstract

**Background:**

This study systematically reviews the literature and performs a meta-analysis to evaluate and compare the intra-operative outcomes, clinical efficacy, safety, and cost of alternative-level and all-level laminoplasty.

**Methods:**

A systematic review is conducted according to the PRISMA guidelines. Searches are performed in PubMed, Cochrane Library, OVID, and Embase databases from inception to August 2024, using search terms “laminoplasty” AND “all OR skip OR alternative.” Data extraction and risk-of-bias assessment are conducted independently by two researchers using the Newcastle-Ottawa Scale. Statistical analysis is performed with RevMan 5.4.

**Results:**

Four retrospective Chinese studies (337 patients: 176 alternative-level, 161 all-level) meet the criteria. Meta-analysis shows no significant difference in intra-operative outcomes: operative time (*P* = 0.23) and blood loss (*P* = 0.11). Clinical efficacy, measured by Japanese Orthopaedic Association(JOA) Score (*P* = 0.08), JOA recovery(*P* = 0.08), and Visual Analog Scale (*P* = 0.26), also shows no significant difference. Similarly, safety outcomes, including complications(*P* = 0.64), C5 palsy(*P* = 1.00), and axial symptoms(*P* = 0.57), are comparable between the two fixation methods. Cervical sagittal parameters are also equivalent: Cervical Curvature Index (*P* = 0.18) and overall range of motion (*P* = 0.29). However, alternative-level laminoplasty demonstrates lower cost (*P* < 0.00001) and is inferior in cervical canal outcomes, including anterior–posterior diameter (*P* = 0.01), Pavlov ratio(*P* = 0.007) and open angle (*P* < 0.00001).

**Conclusion:**

Alternative-level laminoplasty matches all-level fixation in operative efficiency, neurological recovery, and complication rates while substantially reducing implant costs. Its slightly lesser canal expansion does not translate into inferior clinical outcomes. Evidence strength is limited by the small number of single-center retrospective studies from one country. Multi-center randomized trials in other countries are needed to confirm generalizability.

## 1 Introduction

Cervical spondylotic myelopathy(CSM) can lead to neurological dysfunction, severely impacting patients' activities of daily living and quality of life, and placing a heavy burden on patients and society (1–3). Posterior cervical laminoplasty is widely accepted as an effective treatment for multilevel CSM (4–6). In the early stage, sutures were used to fix the opened lamina, but their limited stability allowed the laminae to reclose over time, resulting in recurrent cord compression and compromised outcomes (7–9). The use of mini titanium plates has effectively addressed this issue. Titanium plates provide rigid postoperative support and maintain long-term laminar expansion (10).

In unilateral open-door laminoplasty, two principal mini-plate fixation strategies are used: all-level and alternative-level. Given the high cost of mini titanium plates, whether alternative-level fixation can match all-level fixation in efficacy and safety remains unclear.

This study systematically reviews the existing literature and uses a meta-analysis to compare the effectiveness and safety of skip fixation vs. continuous fixation of mini titanium plates in the treatment of multi-segmental cervical spondylotic myelopathy.

## 2 Methods

This study followed the 2020 Preferred Reporting Items for Systematic Reviews and Meta-Analyses (PRISMA) statement.

### 2.1 Study selection

A systematic review is performed on Pubmed, Cochrane Library, OVID and Embase from inception to August, 2024. The query used in the search is designed to include comprehensive literature. The search words are: “laminoplasty” AND “all OR skip OR alternative”.

### 2.2 Inclusion and exclusion criteria

Articles were included according to the following criteria:

(1) **Patients:** patients diagnosed with cervical spondylotic myelopathy;
(2) **Intervention:** Patients underwent alternative-level laminoplasty;
(3) **Comparison:** Patients underwent all-level laminoplasty;
(4) **Outcomes:** Studies report at least one of the following outcomes: (I) intraoperative outcomes: operative time, blood loss (II) safety: complications, C5 palsy, axial symptoms (III) clinical efficacy follow-up: Japanese Orthopaedic Association Score(JOA), JOA recovery, Visual Analog Scale(VAS) (IV) sagittal parameters: Cervical Curvature Index (CCI), Range of Motion(ROM)(V)cervical canal outcomes: Anterior–posterior diameter(APD), Pavlov ratio, open angle and cost.

### 2.3 Exclusion criteria

(1) Patients are diagnosed with cervical deformity, tuberculosis, tumor, infection.
(2) Animal or cadaver experiment
(3) Conference abstracts, case series, case reports, and technical notes
(4) Studies without included outcomes.

### 2.4 Study selection and data extraction

Two authors independently screened titles/abstracts and full texts against the eligibility criteria. The entire process was supervised by the corresponding author, Liu, who resolved any discrepancies.

Data extraction was also conducted independently by two researchers, with the extracted data subsequently entered into statistical software for analysis. The extracted data included key characteristics of the included studies, such as the first author, publication year, study design, sample size, and outcomes.

### 2.5 Evaluation of risk of bias

The Newcastle–Ottawa Scale was applied to evaluate observational studies. Disagreements were resolved by consensus.

### 2.6 Statistical analysis

The data were analyzed using RevMan 5.4 software. Continuous outcomes were assessed using Mean Difference (MD) with a 95% confidence interval (CI), while dichotomous outcomes were evaluated using the Odds Ratio (OR) and corresponding 95% CI. Standard Mean Difference (SMD) with a 95% confidence interval (CI) is applied in cost analysis due to difference in currency. A *P*-value of less than 0.05 was considered statistically significant. Heterogeneity among the included studies was assessed using the Q-test (*χ*²) and *I*² statistics. If the *P*-value was greater than 0.05 and *I*² < 50%, it indicated no significant statistical heterogeneity, and a fixed-effects model was applied. Conversely, if the *P*-value was less than 0.05 and *I*² > 50%, significant heterogeneity was present, and a random-effects model was employed.

## 3 Results

### 3.1 Study selection

**Figure 1:**
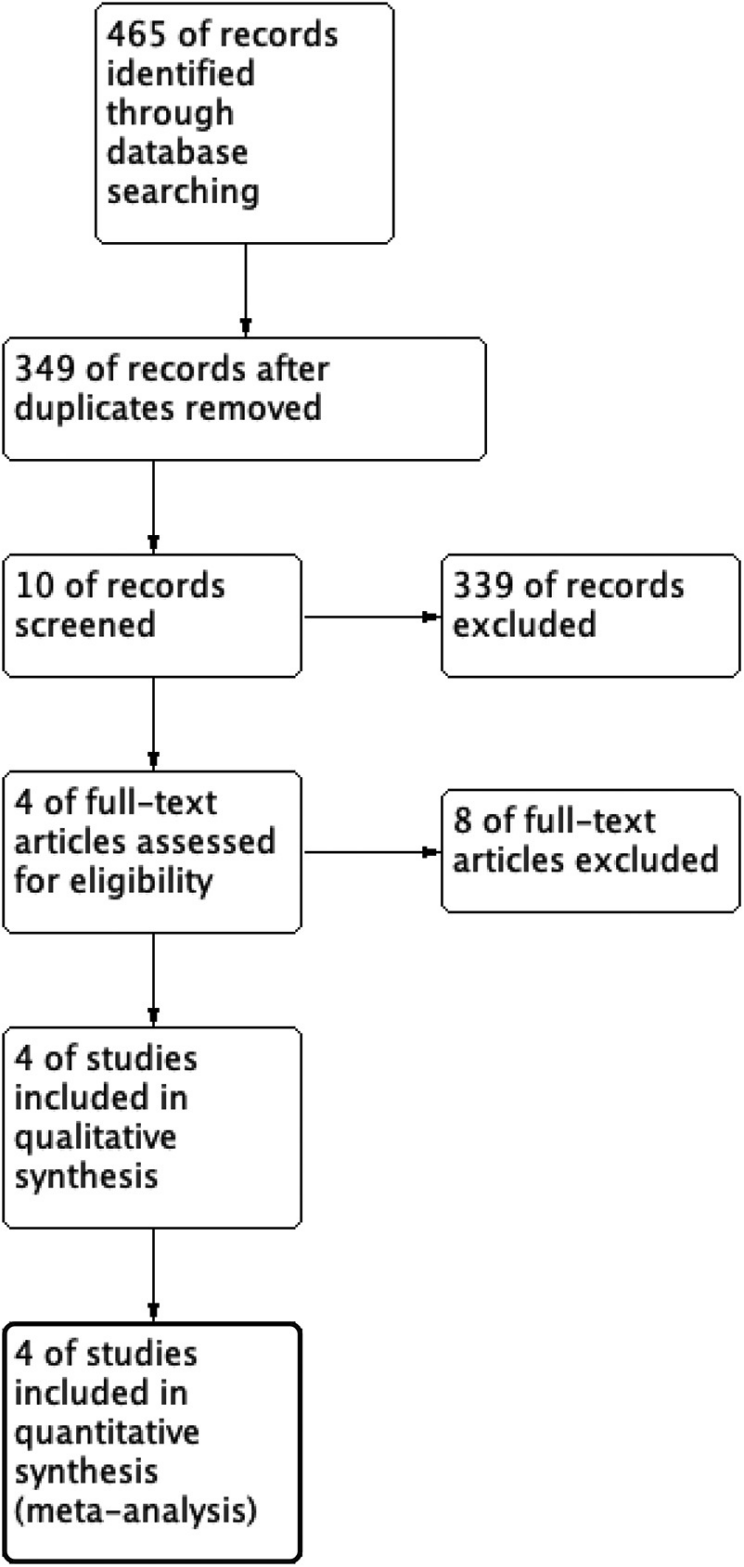
Flow diagram of studies selection.

The initial search included 465 studies. Excluding duplicates, 349 articles were screened by title and abstract. After selection, Four studies met the inclusion criteria for data analysis. The study selection flow chart is shown in Figure 1. The four eligible articles included four comparison groups, with a combined 176 patients who underwent alternative-level laminoplasty, 161 who underwent all-level laminoplasty. Four studies are retrospective studies. The characteristics of included studies are shown in Table 1.

**Table 1:** Characteristic of included studies.

Study	Study type	Sample size		Surgical details	
		Group A^a^	Group B^b^	Group A	Group B
Wang et al. (13)	Retrospective study	51	32	C3, C5 and C7 plate fixation	C3–C7 plate fixation
Yang et al. (12)	Retrospective study	33	34	C3, C5 and C7 plate fixation	C3–C7 plate fixation
Zhang et al. (11)	Retrospective study	38	53	C3, C5 and C7 plate fixation	C3–C7 plate fixation
Liu et al. (14)	Retrospective study	54	42	C4 and C6 plate fixation	C3–C6 plate fixation

^a^ Alternative-level group.

^b^ All-level group.

### 3.2 Risk of bias

The results of the quality evaluation of the observational studies are listed in Table 2. The four included studies exhibit overall good methodological quality. Zhang 2020 and Yang 2019 receive the score of 9. Wang 2014 and Liu 2024 each scored 8.

**Table 2:** Risk of bias assessment using the Newcastle–Ottawa scale for observational studies.

Study	Selection				Comparability		Outcome			Total scores
	Exposed Cohort	Non-exposed Cohort	Ascertainment of exposure	Outcome of interest	The most important factor	Additional factor	Assessment of outcomes	Length of follow up	Adequacy of follow up	
Wang et al. (13)	★	★	★	★	★	★		★	★	8
Yang et al. (12)	★	★	★	★	★	★	★	★	★	9
Zhang et al. (11)	★	★	★	★	★	★	★	★	★	9
Liu (14)	★	★	★	★	★	★		★	★	8

### 3.3 Intraoperative outcomes

#### 3.3.1 Operative time

**Figure 2:**
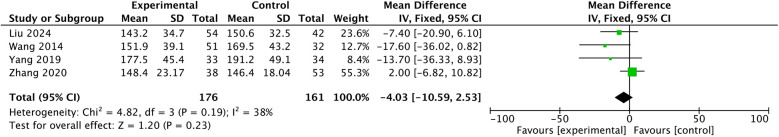
Comparison in operative time.

Four studies report operative time (11–14). All four studies show comparable operative times. The pooled analysis of fixed model confirm this result [MD: −4.03, 95% CI: (−10.59, 2.53), *P* = 0.23; heterogeneity Chi^2^ = 4.82, df = 3, *P* = 0.19, *I*^2^ = 38%], shown in Figure 2.

#### 3.3.2 Blood loss

**Figure 3:**
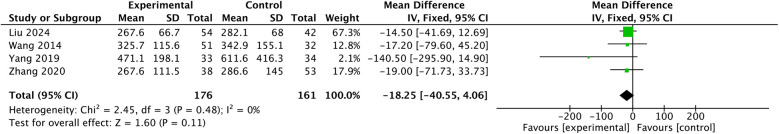
Comparison in blood loss.

Four studies report blood loss (11–14). They all report similar blood loss and pool-analysis of fixed model report same results[MD: −18.25, 95% CI: (−40.55, 4.06), *P* = 0.11; heterogeneity Chi^2^ = 2.45, df = 3, *P* = 0.48, *I*^2^ = 0%], shown in Figure 3.

### 3.4 Cost

**Figure 4:**
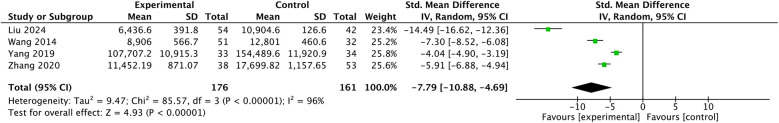
Comparison in cost.

Four studies report cost (11–14). They all report less cost in alternative-level group and pool-analysis of random model report same results [SMD: −7.79, 95% CI: (−10.88, −4.69), *P* < 0.00001; heterogeneity Chi^2^ = 85.57, df = 3, *P* < 0.00001, *I*^2^ = 96%], shown in Figure 4.

### 3.5 Clinical efficacy follow-up

#### 3.5.1 JOA

**Figure 5:**
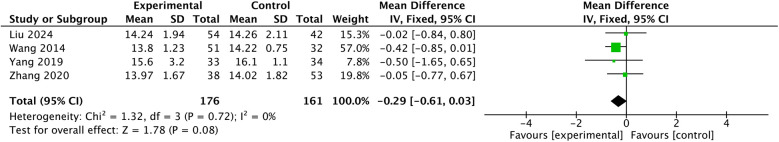
Comparison in JOA.

Four studies report JOA (11–14).They all show similar JOA at final follow-up and pool-analysis of fixed model report same results[MD: −0.29, 95% CI: (−0.61, 0.03), *P* = 0.08; heterogeneity Chi^2^ = 1.32, df = 3, *P* = 0.72, *I*^2^ = 0%], shown in Figure 5.

#### 3.5.2 JOA recovery

**Figure 6:**
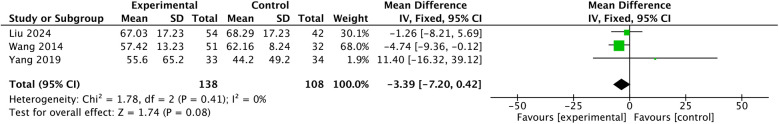
Comparison in JOA recovery.

Wang 2014 reports less JOA recovery. Yang 2019 and Liu 2024 report similar JOA recovery at final follow-up. Pool-analysis of fixed model reports no difference in JOA recovery. [MD: −3.39, 95% CI: (−7.2, 0.42), *P* = 0.08; heterogeneity Chi^2^ = 1.78, df = 2, *P* = 0.41, *I*^2^ = 0%],shown in Figure 6.

#### 3.5.3 VAS

**Figure 7:**
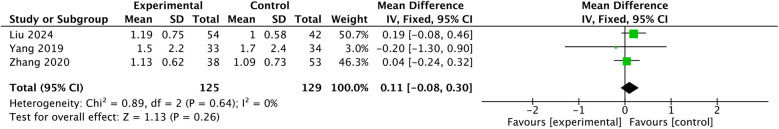
Comparison in VAS.

All studies report no difference in VAS (11–14), and pool-analysis of fixed model report no difference in VAS [MD: 0.11, 95% CI: (−0.08, 0.3), *P* = 0.26; heterogeneity Chi^2^ = 0.89, df = 2, *P* = 0.64, *I*^2^ = 0%], shown in Figure 7.

### 3.6 Safety outcomes

#### 3.6.1 C5 palsy

**Figure 8:**
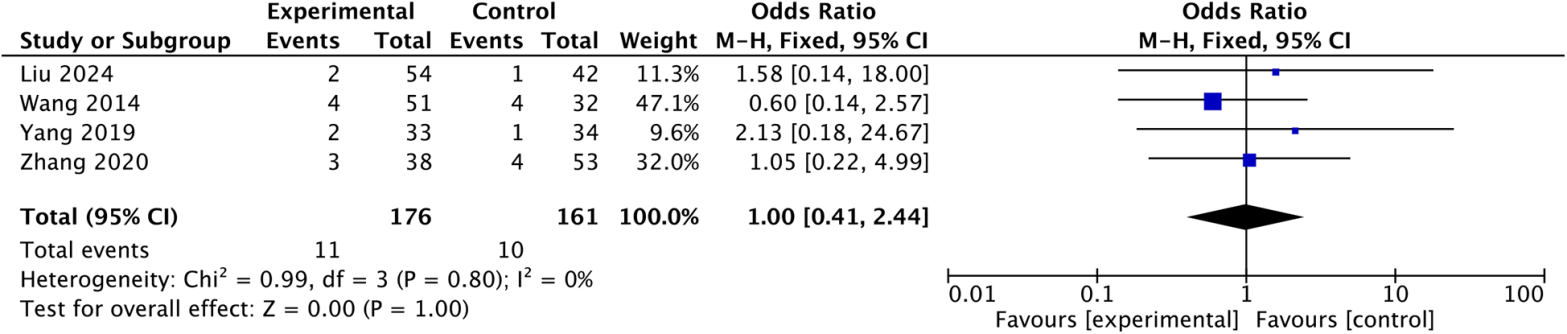
Comparison in C5 palsy.

Four studies report comparison in C5 palsy (11–14). Pool-analysis of fixed model report no difference in C5 palsy [OR: 1.00, 95% CI: (0.41, 2.44), *P* = 1.00; heterogeneity Chi^2^ = 0.99, df = 3, *P* = 0.8, *I*^2^ = 0%], shown in Figure 8.

#### 3.6.2 Axial symptoms

**Figure 9:**
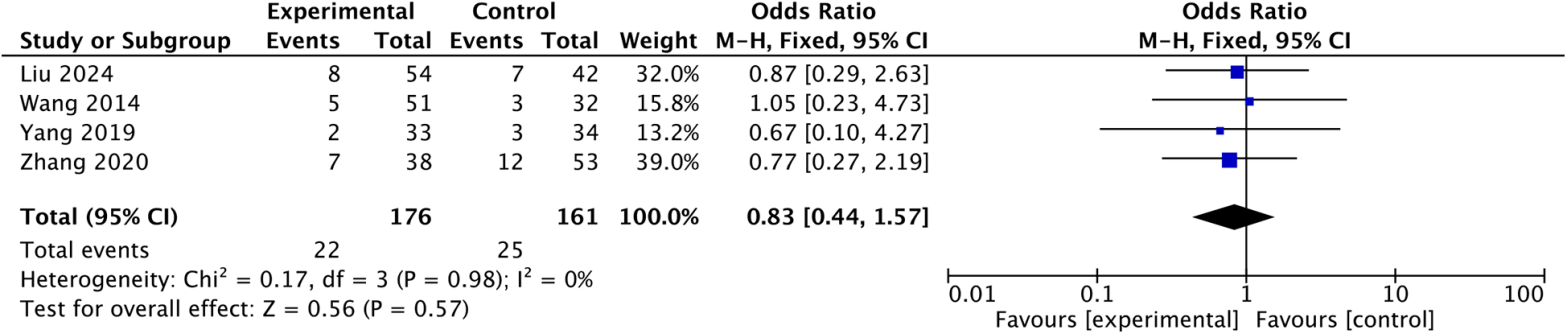
Comparison in axial symptoms.

Four studies report comparison in axial symptoms (11–14). Pool-analysis of fixed model report no difference in axial symptoms [OR: 0.83, 95% CI: (0.44, 1.57), *P* = 0.57; heterogeneity Chi^2^ = 0.17, df = 3, *P* = 0.98, *I*^2^ = 0%], shown in Figure 9.

#### 3.6.3 Complications

**Figure 10:**
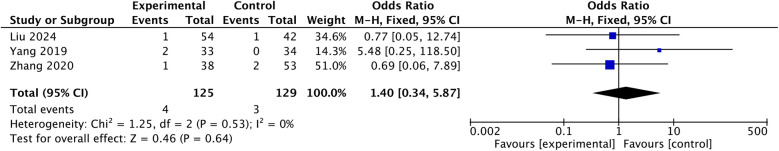
Comparison in complications.

Three studies report no difference in complications (11, 12, 14), and the pooled fixed-model analysis showed no difference in complication rates [OR: 1.40, 95% CI: (0.34, 5.87), *P* = 0.64; heterogeneity Chi^2^ = 1.25, df = 2, *P* = 0.53, *I*^2^ = 0%], shown in Figure 10.

### 3.7. Sagittal radiographic parameters

#### 3.7.1 CCI%

**Figure 11:**
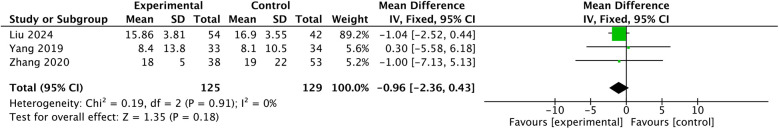
Comparison in CCI.

Three studies report no difference in CCI% (11, 12, 14),and pool analysis of fixed model indicates similar results. [MD: −0.96, 95% CI: (−2.36, 0.43), *P* = 0.18; heterogeneity Chi^2^ = 0.19, df = 2, *P* = 0.91, *I*^2^ = 0%], shown in Figure 11.

#### 3.7.2 ROM

**Figure 12:**
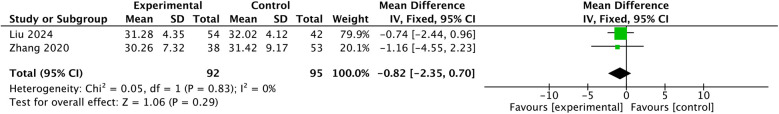
Comparison in ROM.

Two studies report ROM (11, 14). Pool-analysis of fixed model report no difference in ROM [MD: −0.82, 95% CI: (−2.35, 0.7), *P* = 0.29; heterogeneity Chi^2^ = 0.05, df = 1, *P* = 0.83, *I*^2^ = 0%], shown in Figure 12.

### 3.8 Cervical canal outcomes

#### 3.8.1 APD

**Figure 13:**
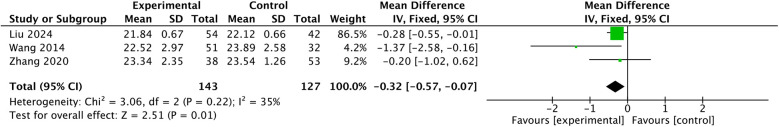
Comparison in APD.

Liu 2024 and Wang 2014 report less APD in alternative-level group. Zhang 2020 reports no difference in APD. Pool-analysis of fixed model indicates less APD in alternative-level group [MD: −0.32, 95% CI: (−0.57, −0.07), *P* = 0.01; heterogeneity Chi^2^ = 3.06, df = 2, *P* = 0.22, *I*^2^ = 35%], shown in Figure 13.

#### 3.8.2 Open angle

**Figure 14:**
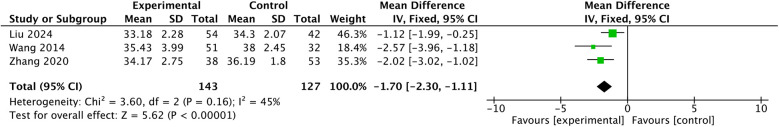
Comparison in open angle.

Three studies report open angle (11, 13, 14). All studies report less open angles in alternative-level group and pool-analysis of fixed model report reports a significantly smaller open angle [MD: −1.7, 95% CI: (−2.3, −1.11), *P* < 0.00001; heterogeneity Chi^2^ = 3.6, df = 2, *P* = 0.16, *I*^2^ = 45%], shown in Figure 14.

#### 3.8.3 Pavlov`s ratio

**Figure 15:**
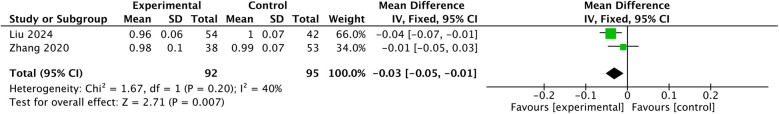
Comparison in Pavlov’s ratio.

Two studies report Pavlov`s ratio (11, 14). Pool-analysis of fixed model report significantly less Pavlov`s ratio in alternative group [MD: −0.03, 95% CI: (−0.05, −0.01), *P* =0.007; heterogeneity Chi^2^ = 1.67, df =1, *P* = 0.2, *I*^2^ = 40%], shown in Figure 15.

## 4 Discussion

Our systematic review and meta-analysis indicates that both alternative-level and all-level laminoplasty achieve comparable clinical outcomes in the treatment of multilevel cervical myelopathy. Postoperative neurological improvements (JOA, JOA recovery) and pain relief(VAS) are similar between the two techniques, with no significant differences observed in functional recovery or complication rates. This suggests that, from a patient-centered clinical perspective, the efficacy and safety of alternate-level laminoplasty are on par with the traditional all-level approach. Both procedures effectively decompressed the spinal cord and led to satisfactory patient outcomes in the follow-up.

Despite these equivalent clinical results, our analysis reveals some radiographic differences between the two techniques. In general, all-level laminoplasty is associated with slightly better preservation of spinal canal expansion on imaging. For example, the all-level group tended to maintain a greater postoperative APD and a higher Pavlov's ratio at final follow-up. Similarly, the lamina open angle is better sustained in the all-level fixation group. At last follow-up, patients who underwent all-level laminoplasty had a marginally larger open angle compared to those with alternate-level fixation. These findings suggest that using plates at every level provides a more rigid and enduring support to keep the laminae open, thereby preventing the minor loss of canal expansion that can occur at alternative levels. In the alternate-level technique, the segments without a supporting plate experienced a slight inward settling of the lamina (11). These radiographic trends are consistent with the patterns reported in individual comparative studies (11, 13, 15) and highlight a biomechanical distinction: all-level fixation confers greater long-term stability to the expanded laminae.

The clinical relevance of these radiographic differences, however, appears limited within the follow-up periods reported. Although the alternate-level group showed statistically significant reductions in canal metrics (APD, Pavlov's ratio, open angle) over time, these changes were small in magnitude and did not translate into worse clinical outcomes in the studies analyzed. All included studies reported no significant between-group differences in postoperative neurological status or disability scores, despite the imaging disparities. In practical terms, even though all-level laminoplasty preserved slightly more canal space, the absolute canal dimensions achieved by alternate-level laminoplasty remained sufficient to prevent spinal cord recompression. Therefore, the statistically significant radiographic advantages observed with all-level fixation should be interpreted with caution—they may represent subclinical differences that have little impact on patient recovery. All-level laminoplasty does indeed provide more sustained spinal canal enlargement radiologically, but this incremental radiological advantage does not translate into significant clinical benefit. While using more implants results in higher surgical costs, patient subjective outcomes do not improve correspondingly.

Complications are categorized as overall complications, C5 palsy, and axial pain. There is no significant difference in the incidence of these complications between the two methods. The three analysis show low heterogeneity, indicating that the findings are robust. Although the absolute event count was small, the current evidence suggests that reducing plate density does not increase peri-operative or long-term complication rates. Nevertheless, large-scale, prospective studies with standardized complication definitions are required to verify equivalence within narrower confidence intervals.

In terms of sagittal parameters, CCI represents the physiological lordosis of the cervical spine and is closely related to the posterior spinous muscle complex. Since there is no difference in the degree of exposure between the two fixation techniques, final follow-up results showed no significant difference in CCI. Modified surgical techniques, such as C3 laminectomy (16, 17) or preserving muscles complex technique (18, 19), can further maintain the physiological lordosis of the cervical spine.

This meta-analysis incorporates four studies, all conducted at medical centers in China. Although this provides a degree of uniformity in patient pathology, surgical techniques, and peri-operative protocols, it also constrains the external validity of our findings. Regional variations in surgical preferences—such as hinge placement and titanium plate selection—along with differences in implant pricing structures, reimbursement policies, and patient anatomical characteristics, may affect both the feasibility of alternative-level fixation and its radiographic or clinical outcomes (e.g., baseline sagittal alignment parameters). Therefore, the pooled results presented here should be considered hypothesis-generating when applied outside the Chinese clinical context. Prospective, multicenter investigations across diverse healthcare systems are needed to determine whether these conclusions can be generalized globally.

In our meta-analysis, several outcomes—most notably cost (*I*^2^ = 96%)—demonstrated a high level of statistical heterogeneity. The cost heterogeneity likely stems from variations among hospitals and regions, as well as differences in the study time window. Radiological parameters, such as open angle (*I*^2^ = 45%) and, to a lesser extent, APD (*I*^2^ = 35%), may show heterogeneity because of differences in hinge placement, plate specifications, and follow-up duration. We employed random-effects models for all outcomes with *I*^2^ > 50%, yet multi-center prospective studies are still required to reduce this uncertainty further.

Limitations: (1) The number of included studies is limited, with only Four studies incorporated into the meta-analysis, and the sample size is relatively small, resulting in several outcomes with *P* values between 0.05 and 0.10 and potential corresponding risk of type II error. Four studies are all retrospective studies. High-quality randomized controlled trials can provide a higher level of evidence. (2) All eligible studies are from China. Although the enrolled populations are relatively homogeneous, the findings may not be generalizable to regions with different surgical training paradigms, implant markets, or patient demographics. Future randomized controlled trials or well-designed prospective cohort studies from diverse geographic settings are needed to further validate these conclusions. (3) This study only included publicly published literature in English, lacking literature in other languages. (4) Some of the included indicators, such as ROM and Pavlov's ratio, have an insufficient number of studies, with only two studies reporting relevant content. This may also introduce bias.

## 5 Conclusion

Alternative-level laminoplasty is comparable to all-level laminoplasty in intraoperative outcomes (operative time, blood loss), safety (complications, C5 palsy, axial symptoms), clinical efficacy follow-up(JOA, JOA recovery, VAS) and sagittal parameters(CCI, ROM). Alternative-level is inferior in cervical canal outcomes (APD, Pavlov`s ratio, open angle). It is superior in cost. Four included studies are all single-center retrospective investigations from China, the evidence level is limited, and regional variations in surgical preferences, implant pricing, and patient anatomy may restrict external generalizability. Accordingly, surgical decisions should be individualized, balancing cost savings against the need for adequate decompression, and high-quality multicenter randomized controlled trials across diverse health-care systems are urgently needed to validate these findings.

## Data Availability

The original contributions presented in the study are included in the article/Supplementary Material, further inquiries can be directed to the corresponding author.
